# Neuro-Symbolic Class-Contrast Evidence Audit for Reliable Cross-Subject Wearable Activity Recognition

**DOI:** 10.3390/s26144390

**Published:** 2026-07-10

**Authors:** Qiang Li, Zhirong Qu, Meng Yan, Xiaohong Zhang

**Affiliations:** 1 School of Big Data and Software Engineering, Chongqing University, Chongqing 401331, China; liqiang@cqwu.edu.cn (Q.L.); xhongz@cqu.edu.cn (X.Z.); 2 School of Mathematics and Artificial Intelligence, Chongqing University of Arts and Sciences, Chongqing 402160, China; 202506025049@stu.cqwu.edu.cn

**Keywords:** wearable sensors, human activity recognition, rule-guided audit, class-contrast retrieval, selective prediction, knowledge provenance chain

## Abstract

Reliable wearable activity recognition requires not only a class label but also an auditable indication of whether that label is supported by historical sensor evidence. We present CC-NSIEA, a label-preserving neural-plus-rule-based class-contrast evidence audit for cross-subject wearable activity recognition. A Temporal Residual Perception Network supplies the sole activity label, posterior probabilities, and a normalized temporal embedding. A read-only Training-Subject Evidence Memory retrieves global, predicted-class, and competing-class records. A rule-based Evidence Consistency Audit combines data validity, dynamic/static motion coherence, retrieval support, and class separation. When first-round evidence is insufficient, Class-Contrast Evidence Refinement performs one deterministic contrast between the predicted class and the strongest posterior competitor; the audit cannot change the neural label. The term neuro-symbolic is used only in this restricted architectural sense: a neural predictor is coupled to explicitly represent deterministic predicates and a finite rule-based controller; the method does not perform symbolic inference, theorem proving, or knowledge-graph reasoning. On five subject-disjoint outer folds of the UCI HAR official training partition, the shared perception model achieved 90.13% accuracy and 90.55% macro-F1 across 7352 out-of-fold windows from 21 subjects. Relative to a matched dynamic deterministic controller, CC-NSIEA increased Error AUPRC from 0.423802 to 0.433057 and reduced AURC from 0.035941 to 0.035913. The 10,000-resample subject-cluster bootstrap interval for the AUPRC difference was [0.001595, 0.019547]. CC-NSIEA provides an evidence-centered complement to confidence-based reliability estimation.

## 1. Introduction

Wearable inertial sensing supports activity-aware services in rehabilitation, assisted living, fitness monitoring, and context-adaptive interfaces. The present study evaluates this reliability-auditing setting on the publicly available UCI Human Activity Recognition Using Smartphones benchmark [1]. In these applications, a classifier is frequently evaluated by average accuracy, yet this aggregate quantity is not sufficient for operational use. A prediction made for a previously unseen subject may be correct, incorrect but low-confidence, or incorrect despite high posterior confidence. The last case is particularly consequential because a downstream system has no obvious signal that additional review or context is needed. Cross-subject activity recognition is therefore naturally a two-output problem: the system should provide an activity label and an independently inspectable estimate of whether that label is well supported by available evidence.

Deep temporal models have reduced dependence on handcrafted features in wearable HAR by learning local patterns and longer temporal dependencies directly from inertial sequences [2,3,4,5,6]. Residual architectures and attention-style pooling are useful for extracting compact sequence-level representations [7]. However, a strong representation learner does not in itself explain why a specific prediction should be trusted. Calibration, selective prediction, and error-detection research show that posterior confidence and entropy can be valuable reliability signals, but they need not distinguish every high-confidence error or reveal which evidence contradicts a prediction [8,9,10,11,12,13]. For wearable deployment, a reliability signal is more useful when it can be traced to sensor evidence, explicit checks, and a documented stopping decision.

Retrieval-augmented and self-reflective language model systems motivate a general design principle: obtain external evidence when the available state is inadequate, verify whether that evidence is sufficient, and avoid unnecessary additional retrieval [14,15,16]. This principle is useful beyond text generation, but direct transplantation is inappropriate here. The task is not natural-language answering, no external text corpus is required, and an evidence mechanism should not be allowed to replace a sensor classifier with an unvalidated generated label. We instead treat retrieval as a read-only operation over historical sensor representations generated from training subjects. The controller is deliberately constrained to enrich evidence and estimate risk, not to generate, revise, or rank the final activity label.

Rule-guided neural auditing provides a second motivation. Rather than relying on opaque post hoc narratives, explicit deterministic checks can record whether an input is valid, whether broad motion characteristics agree with a predicted activity group, whether nearby historical records support the prediction, and whether the closest competing class remains insufficiently separated [17,18,19,20,21]. In this manuscript, neuro-symbolic is used only as a narrow implementation descriptor for coupling neural perception with explicit predicate checks and finite control rules; it is not a claim of general symbolic inference, logical reasoning, or knowledge-graph reasoning. Such checks are intentionally modest: they are not claimed to encode a complete ontology of human behavior. Their purpose is to expose auditable conditions that accompany a neural prediction. This design permits a provenance record that contains evidence identifiers, numerical support values, rule outcomes, and stopping reasons, without claiming token attribution, hidden chain-of-thought access, or human-expert scores.

Accordingly, this study addresses the following question: given a fixed cross-subject neural activity prediction, can a deterministic class-contrast evidence audit better identify predictions that are likely to be wrong than an otherwise matched dynamic audit controller? The study does not formulate anomaly detection, streaming event propagation, medical diagnosis, or legal reasoning as evaluated tasks. It is restricted to six-class wearable activity recognition on UCI HAR and to post hoc error-risk auditing of the fixed neural output.

The contributions are fourfold. First, we formulate CC-NSIEA as a label-preserving separation of neural perception and evidence auditing. Second, we introduce a deterministic class-contrast refinement policy that explicitly contrasts evidence for the neural prediction with evidence for its strongest competing class. Third, we specify an evidence sufficiency controller with finite, ordered stopping conditions and a structured Knowledge Provenance Chain (KPC). Fourth, we evaluate the method under a four-way subject-disjoint protocol with separate model selection and audit calibration roles, and quantify controller differences by subject-cluster bootstrap rather than treating overlapping windows as independent samples [22].

## 2. Related Work

Wearable HAR has evolved from manually engineered inertial features to learned multichannel temporal representations. DeepConvLSTM, convolutional/recurrent comparison studies, and ensemble LSTM models demonstrate that raw or lightly processed wearable sequences can support end-to-end feature learning [2,3,4]. Subsequent time-series architectures, including residual and inception-style models, reinforce the value of local temporal filters, residual optimization, and compact sequence embeddings [5,6,7]. These works primarily target activity classification. CC-NSIEA uses a temporal residual classifier as its perception backbone, but its contribution lies after the label is produced: it evaluates the evidential support of that label without revising it.

Prediction confidence, entropy, ensemble uncertainty, and selective classification offer complementary tools for identifying potentially unreliable decisions [8,9,10,11,12,13]. Confidence-based scores are often strong global ranking signals, while selective prediction evaluates risk after rejecting or deferring high-risk examples. Our evaluation follows this distinction. Accuracy and macro-F1 assess the shared perception model once. Error AUROC, Error AUPRC, AURC, and top-risk error concentration assess whether an audit score identifies incorrect fixed labels. This separation avoids an invalid interpretation in which a risk-audit module is credited with classification gains that it cannot produce under a label-preserving constraint.

Retrieval-augmented generation [14], self-reflective retrieval [15], and reasoning-action frameworks [16] have emphasized evidence acquisition, inspection, and adaptive control for language tasks. We cite these lines of work as conceptual precedents for evidence sufficiency and retrieval verification, not as baselines. Their language-generation objectives, corpora, and evaluation settings differ fundamentally from read-only sensor-memory retrieval. In the present study, the query is a neural embedding, retrieved items are training-subject sensor windows, and the only permitted refinement is a predefined evidence view. This boundary is essential for reproducibility and for preventing an external controller from becoming an unmeasured classifier.

Structured rules and semantic validation are also active topics in IoT-oriented research. Cimino et al. [17], for example, study contrastive rule representations and ranking for IoT trigger-action generation. More broadly, neural-symbolic research examines couplings of learned representations with explicit symbolic constraints when an output must be checked rather than accepted solely because a neural score is high [18,19]. CC-NSIEA adopts only a constrained rule-guided form of this design: four deterministic checks provide interpretable audit components, and their records are retained in the KPC. The method does not execute a symbolic inference engine, ontology reasoning, or knowledge-graph reasoning.

Finally, explainability should be distinguished from traceability. Local attribution methods can help explain learned predictors [20,21], but the present method does not implement SHAP, token attribution, or natural-language rationale generation. Its explainability claim is limited to a structured provenance record. Recent work on controlled LLM behavior and contrastive control in code-oriented systems [23,24] further motivates the need to separate the effect of a controller from the effect of the base model or retrieval policy. We therefore include a controller-equivalence diagnostic rather than assume that an LLM-planned action is beneficial merely because it is generated by an LLM.

## 3. Method

### 3.1. Problem Boundary and Notation

Problem definition. Let x_i_ denote the i-th standardized inertial window and let y_i_ denote one of six activities. The perception model f_θ_ maps x_i_ to a normalized embedding z_i_ and a class-posterior vector p_i_. The fixed neural label is the highest posterior class. CC-NSIEA receives the input, embedding, posterior vector, and neural label, but does not receive or use the ground-truth activity during inference. Its output is the unchanged neural label, an audit-risk score, and a KPC record. The label-preservation invariant is explicit: no retrieved neighbor, rule result, or controller action may change the neural label.

(1) $$x_{i}\in R^{9\times 128},\;y_{i}\in Y,\;f_{\theta }(x_{i})=(z_{i},p_{i}).$$

(2) $$z_{i}\in R^{128},\;p_{i}\in [0,1{]}^{\left|Y\right|},\;{\hat{y}}_{i}=\arg\underset{y\in Y}{\max}\;p_{i}(y).$$

### 3.2. Temporal Residual Perception Network

**Figure 1:**
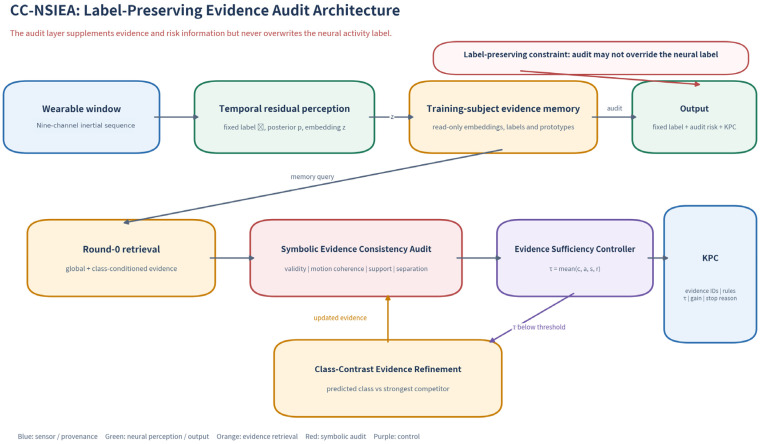
Overall CC-NSIEA architecture. The audit pipeline enriches evidence and risk information but cannot overwrite the neural label.

The backbone begins with a one-dimensional convolutional stem that maps the nine-channel sequence to 64 latent channels. Four residual temporal blocks with dilation rates 1, 2, 4, and 8 then enlarge the temporal receptive field while preserving an identity path. Each block uses two convolutional layers, group normalization, GELU activation, and dropout. Attention pooling converts the temporal feature map into a sequence-level vector, followed by a two-layer projector that produces a 128-dimensional embedding. The classifier head operates on the pre-normalized projection, whereas evidence retrieval uses the L2-normalized embedding. The network is trained with cross-entropy only; no contrastive loss, label override loss, or controller reward is used. This design makes the label-producing component explicit and makes the evidence representation available for a separate audit layer. Figure 1 presents the overall architecture and the hard label-preservation boundary.

### 3.3. Training-Subject Evidence Memory

For each outer fold, memory is constructed only after the selected perception model has been trained. It contains the normalized embeddings, activity labels, source indices, and subject identifiers of records from the model-train and model-selection subjects. Outer-test and audit-calibration records are excluded from memory construction. For each class y, TSEM also stores a normalized class prototype μ_y_ obtained by averaging its memory embeddings. Given query z_i_ and strongest competing class y_i_^c^, retrieval uses the cosine distance defined below. The initial round retains the five nearest global neighbors and five nearest neighbors from each of the predicted and competing classes.

(3) $$d(z_{i},z_{j})=1-z_{i}^{⊤}z_{j}.$$

### 3.4. Rule-Based Symbolic Evidence Consistency Audit (SECA)

The audit computes four bounded components. Neural confidence is the largest posterior probability for the fixed neural label. Retrieval support a_i_ is the fraction of initial global neighbors whose label equals ŷ_i_. Class separation s_i_ combines a nearest-neighbor separation term and a prototype-separation term. Let d_p_ and d_c_ be the nearest predicted-class and competing-class distances. The local separation uses a clipped normalized distance margin between the closest predicted-class and competing-class records. A corresponding prototype separation uses the distances to μ_ŷ_ and μ_yc_. The final separation score is their arithmetic mean. The rule-consistency component r_i_ averages two deterministic checks. The data-validity rule verifies finite values and bounds the maximum absolute standardized sensor value by 30. The motion-group rule derives root-mean-square body-motion energy from the three body-acceleration channels; a sigmoid score relative to a memory-fitted dynamic/static boundary assesses whether the predicted label belongs to a compatible motion group. Dynamic labels are walking, walking upstairs, and walking downstairs, whereas sitting, standing, and laying form the static group. In this restricted use, symbolic denotes explicitly represented deterministic predicates and rules, not a separate symbolic inference engine. These rules are audit signals only; they are not classifiers and do not determine ŷ_i_.

### 3.5. Evidence Sufficiency Controller and Class-Contrast Refinement (ESC)

**Figure 2:**
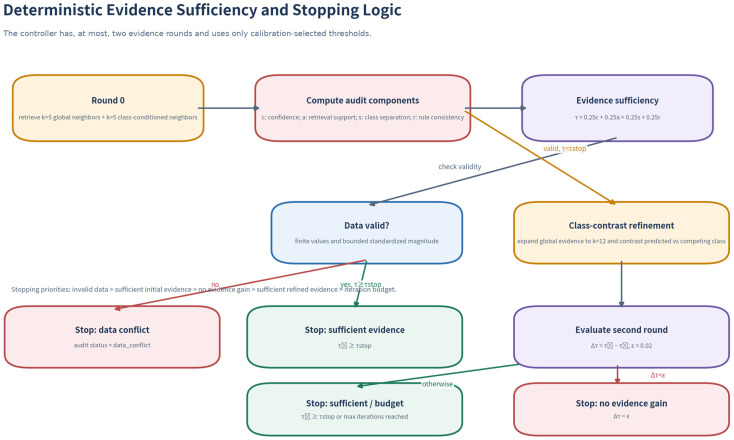
Deterministic evidence sufficiency, class-contrast refinement, and ordered stopping logic. Colored boxes denote audit states and stopping outcomes, and arrows indicate deterministic control flow and conditional transitions.

The four components form the transparent sufficiency score shown below. Equal weights are fixed in the configuration. The threshold τ_stop_ is selected only on audit-calibration subjects from the predeclared grid {0.55, 0.60, 0.65, 0.70, 0.75} by maximizing calibration error-detection AUPRC. The outer-test labels are never inspected for this choice. The audit risk is the complement of the final-round sufficiency score. A high q_i_ therefore indicates limited confidence, weak neighbor agreement, insufficient class separation, rule inconsistency, or a combination of these factors. Figure 2 presents the finite evidence sufficiency and stopping procedure.

(4) $$\tau_{i}=0.25c_{i}+0.25a_{i}+0.25s_{i}+0.25r_{i},\;\;q_{i}=1-\tau_{i}.$$

Class-Contrast Evidence Refinement (CCER). If the input is valid and τ_i_ is below τ_stop_ after round 0, CCER performs exactly one additional evidence round. The global retrieval budget increases from five to 12 neighbors; predicted-class and competing-class views remain class conditioned. CCER emphasizes the contrast between evidence for the neural predicted class and its strongest posterior competitor. It recomputes support, separation, rule consistency, and τ_i_ using the expanded evidence view. The evidence gain is defined below. CCER is deliberately finite. There is no query rewriting, free-form retrieval planning, or repeated iteration until a desired label is obtained.

(5) $$\Delta \tau_{i}=\tau_{i}^{\left(1\right)}-\tau_{i}^{\left(0\right)}.$$

### 3.6. Ordered Stopping and Knowledge Provenance Chain

Ordered stopping logic. The controller uses a maximum of two evidence rounds. First, invalid input terminates immediately with stop reason ‘data_validity_conflict’. Second, an initial τ_i_ ≥ τ_stop_ terminates with ‘sufficient_evidence’. Otherwise, CCER is executed once. After refinement, Δτ_i_ < 0.02 yields ‘no_evidence_gain’ and τ_i_^(1)^ ≥ τ_stop_ yields ‘sufficient_evidence’; all remaining cases terminate with ‘max_iter_reached’. This ordering removes the ambiguity present in open-ended retrieve-reason loops. It also guarantees that the stopping decision is determined by explicit numeric and deterministic rule-based conditions rather than by an unlogged language model judgment. The controller evaluates these conditions in the stated order and terminates as soon as the first applicable condition is satisfied; no later rule is evaluated after termination.

**Figure 3:**
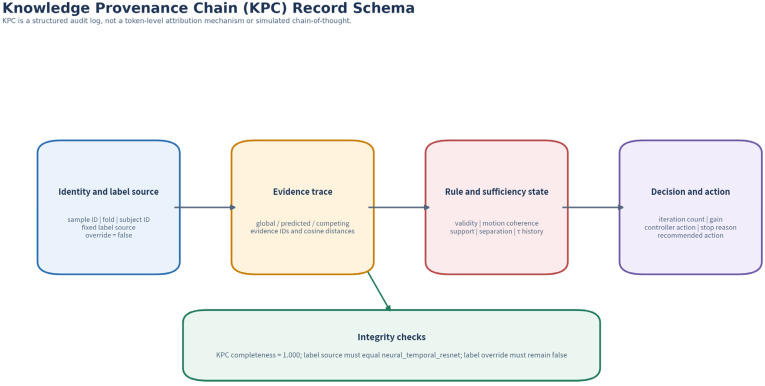
KPC record schema. The record is a structured audit trace, not a token-level attribution mechanism. Colored blocks denote groups of KPC fields, and arrows indicate the recorded audit-trace order.

Knowledge Provenance Chain (KPC). For every outer-test window, κ_i_ records the source index, fold, subject identifier, neural label source, override constraint, predicted label, strongest competitor, confidence, entropy, retrieval rounds, evidence identifiers, cosine distances, rule results, τ history, evidence gain, risk, audit status, controller action, recommended action, and stop reason. Completeness is checked mechanically: all required fields must be present, ‘label_source’ must equal ‘neural_temporal_resnet’, and ‘override_permitted’ must remain false. The KPC is therefore a structured provenance object that can be inspected independently of a narrative explanation. Figure 3 summarizes the fields retained in the KPC record.

## 4. Experimental Protocol

### 4.1. Dataset and Signal Representation

We use the UCI Human Activity Recognition Using Smartphones dataset [1]. The revised protocol deliberately uses only the official training partition. This partition contains 7352 windows from 21 subjects; the official test split and its labels are not used. The six classes are walking, walking upstairs, walking downstairs, sitting, standing, and laying. Each window has 128 time samples. Instead of the 561-dimensional engineered feature vector, the implementation loads three axes each of total acceleration and body acceleration, derives gravity acceleration as total minus body, and concatenates these signals into nine channels. This preserves channel identity and temporal structure. It also avoids the unsupported claim that a text summary of global statistics can recover sensor-axis-specific evidence.

### 4.2. Four-Way Subject-Disjoint Protocol

**Figure 4:**
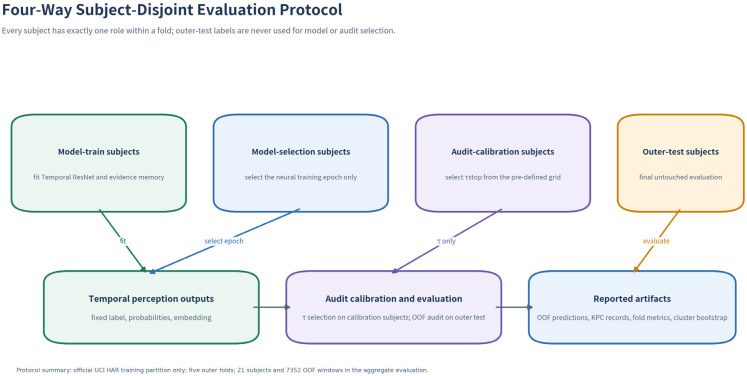
Four-way subject-disjoint protocol. Model selection and audit calibration have separate subject roles. Colored boxes denote subject roles and reported artifacts, and arrows indicate the evaluation flow across model training, model selection, audit calibration and outer testing.

GroupKFold produces five outer folds over subject identities. Within the outer-train subjects of each fold, a predefined search selects a class-coverage-balanced partition into model-train subjects, two model-selection subjects and two audit-calibration subjects. The partition is selected only from subjects and labels before model training; no model score or outer-test label is inspected. Model-train subjects fit the perception model. Model-selection subjects select the training epoch. The model is then retrained on model-train plus model-selection records for the selected number of epochs. Audit-calibration subjects select only τ_stop_. Outer-test subjects are used once for final prediction and audit. This separation is important because a threshold chosen on the final test subjects would inflate error-risk metrics even when the activity classifier itself remains fixed. Figure 4 depicts the four subject roles and the resulting evaluation flow.

### 4.3. Training, Baselines, and Implementation

The Temporal Residual Perception Network uses 64 latent channels, a 128-dimensional embedding, a dropout of 0.10, and dilation rates 1, 2, 4, and 8. The optimizer uses the learning rate and weight-decay settings shown below, a batch size of 128, gradient clipping at 5.0, and Gaussian jitter augmentation with a standard deviation of 0.012 during training. Training is capped at 35 epochs with an early stopping patience of 8 during epoch selection. The final fold model is trained for the selected epoch count. The audit configuration fixes maximum iterations at two, initial global top-k at 5, refined global top-k at 12, class-conditioned top-k at 5, evidence gain epsilon at 0.02, and equal sufficiency weights. All configuration values, split definitions, and execution commands are provided in the released code package.

η = 10^−3^,    λ = 10^−4^.

### 4.4. Metrics, Statistics, and Reproducibility

Baselines and controlled comparisons. We do not label nearest-neighbor voting as “Vanilla RAG”, and we do not use a keyword heuristic such as “Self-RAG.” All reported audit methods share the same frozen neural predictions, memory bank, subject-disjoint protocol, and final labels. Confidence-only uses 1-max p; entropy-only uses predictive entropy; retrieval-only uses retrieval support and separation without rule consistency; rule-only uses the explicit rule signals; D-NSIEA is the original dynamic deterministic action selector; and CC-NSIEA always chooses the class-contrast evidence view when a second round is needed. This matched design isolates the consequence of evidence-action selection rather than mixing different classifiers, corpora, or labels. Table 1 summarizes the shared perception results. Uncertainty intervals for controller comparisons are estimated using 10,000 subject-cluster bootstrap resamples, which resample subjects rather than overlapping windows [22].

## 5. Results

### 5.1. Shared Perception Performance

Across the five outer folds, the shared Temporal ResNet produced 7352 OOF predictions. The aggregate accuracy was 0.901251 and the macro-F1 was 0.905547. The bootstrap 95% interval for accuracy was [0.894314, 0.908192], and the interval for macro-F1 was [0.899589, 0.911973]. The global 15-bin expected calibration error was 0.071352. Per-class performance was strongest for laying and walking downstairs, while the principal confusion was between the two static postures: 309 sitting windows were predicted as standing and 218 standing windows were predicted as sitting. This pattern does not invalidate the classifier; rather, it provides concrete motivation for evidence auditing because static-class boundaries can be uncertain even when the broad motion group is consistent. Per-class results are reported in Table 2.

### 5.2. Risk-Estimator Comparison

Confidence and entropy were strong global error-ranking baselines: confidence obtained Error AUROC 0.832074 and AURC 0.028883, whereas entropy obtained Error AUROC 0.831298 and AURC 0.028983. CC-NSIEA did not surpass confidence in these two metrics and we do not claim otherwise. Its strength is error-focused precision-recall behavior and high-risk concentration. CC-NSIEA attained Error AUPRC 0.433057, compared with 0.390254 for confidence, and the highest top-10% high-risk error rate of 0.479620. Retrieval-only and rule-only scores were weaker than the combined audit. These results support complementarity: neural posterior confidence remains useful for broad ranking, while evidence support, separation, and explicit consistency checks help concentrate likely errors among the cases that the audit flags. The full comparison is reported in Table 3.

### 5.3. Matched Controller Comparison

**Figure 5:**
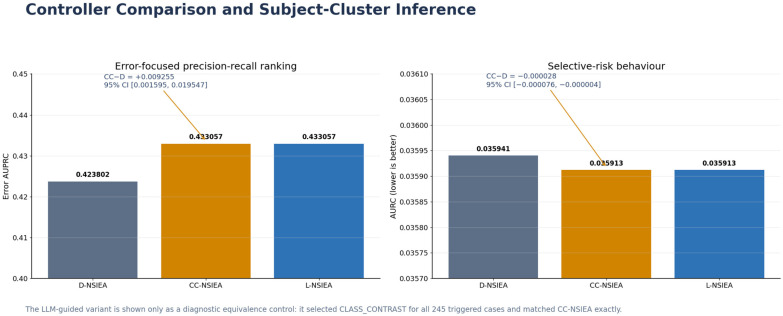
Controller comparison and subject-cluster inference.

D-NSIEA and CC-NSIEA use the same neural model, evidence memory, thresholds, KPC schema, and stopping authority. Their only difference is the second-round evidence action. D-NSIEA selects among predicted-class support, class contrast, motion-group contrast, and prototype contrast by deterministic rules. CC-NSIEA always uses class contrast when a second round is required. CC-NSIEA increased Error AUROC from 0.786100 to 0.786411, Error AUPRC from 0.423802 to 0.433057, and reduced AURC from 0.035941 to 0.035913. Subject-cluster bootstrap yielded an AUPRC difference of 0.009255 with a 95% interval [0.001595, 0.019547]. The AURC difference was −0.000028 with a 95% interval [−0.000076, −0.000004]. Four folds improved AUPRC and one was unchanged; none decreased. The absolute changes are modest and should be interpreted as a targeted improvement in the evidence-audit policy, not as a large activity-recognition gain. Its practical role is limited to the audit queue: with the neural labels held fixed, a higher Error AUPRC means that, across review thresholds, the audit score better ranks windows that are likely to be incorrect. The observed gain is therefore meaningful only as a small, subject-consistent improvement in prioritizing cases for review; it does not demonstrate a change in classification accuracy, top-10% risk concentration, or downstream user outcomes. These results are specific to the present UCI HAR protocol and require confirmation on additional wearable activity-recognition datasets. Table 4 and Table 5 report the matched controller comparison and subject-cluster inference, respectively; Figure 5 visualizes the paired comparison.

### 5.4. Stopping, Provenance, and Controller Diagnostics

CC-NSIEA stopped after the initial round for 7156 windows because the evidence sufficiency threshold was met. It returned ‘no_evidence_gain’ for 104 windows and ‘max_iter_reached’ for 92 windows. The mean number of audit rounds was 1.033324. KPC completeness was 1.000: every OOF record contained the required identity, evidence, rule, sufficiency, and decision fields, and every record preserved ‘label_source = neural_temporal_resnet’ with ‘override_permitted = false’. These checks make it possible to identify whether a high-risk decision resulted from low confidence, weak local support, inadequate class separation, a motion-coherence conflict, or insufficient gain from refinement. Table 6 summarizes the stopping outcomes and KPC completeness checks.

Controller-equivalence diagnostic. We also evaluated a constrained LLM-guided evidence-action planner under the same fixed-label protocol. It was invoked for 245 insufficient-evidence cases and selected ‘CLASS_CONTRAST’ for all 245. A forced deterministic class-contrast controller therefore provided the required causal counterfactual. The two variants matched exactly on all 7352 source-index-aligned OOF windows: activity labels, audit risks, Error AUROC, Error AUPRC, AURC, and top-10% error rate were identical, and the maximum absolute audit-risk difference was zero. The 10,000-resample subject-cluster confidence intervals for all CC-versus-LLM metric differences were [0,0]. The LLM controller required 418.95 s in the paired audit run compared with 34.93 s for the dynamic deterministic controller. Accordingly, it is not part of the final method. This diagnostic prevents an unsupported claim that the observed effect arises from LLM planning.

Label-override diagnostic. As an additional frozen diagnostic analysis, we examined whether an iterative component should be allowed to overwrite neural activity labels. On 2941 held-out predictions, the override variant obtained an accuracy of 0.908875 and a macro-F1 of 0.910346, compared with 0.910915 and 0.912188 for the unmodified baseline. It changed 36 originally correct predictions to wrong labels and corrected 30 originally wrong predictions; McNemar’s exact *p*-value was 0.538583. Because that diagnostic uses a separate legacy hold-out protocol, it is not merged with the main OOF controller comparison. It is included solely to justify the hard label-preservation constraint adopted by CC-NSIEA.

## 6. Discussion and Limitations

The main empirical finding is narrow but useful. A deterministic second-round contrast between the predicted class and the strongest competitor improves the error-focused precision-recall ranking of the audit score relative to a dynamic controller under otherwise matched conditions. The gain is not a new classifier and should not be represented as an accuracy improvement. Rather, it shows that when initial evidence is insufficient, a direct comparison of the two most relevant class hypotheses can be a more stable evidence view than switching among several heterogeneous retrieval emphases. Practically, the gain concerns the ordering of a finite review queue: the audit does not correct labels, but it can make windows selected for review more error-enriched across the precision-recall curve. Because the top-10% error rate is unchanged between D-NSIEA and CC-NSIEA, the current evidence supports improved global precision-recall ranking rather than a claim of a fixed review-budget improvement.

The result also clarifies the role of language models in this setting. A language model may appear attractive as a flexible action planner, but flexibility is not a substitute for causal attribution. Here, the constrained planner collapsed to the same class-contrast action in every invocation. The correct conclusion is not that the LLM failed in an absolute sense; it is that no measurable independent effect remained after a deterministic fixed-action control was introduced. Removing the external API from the final system improves reproducibility, latency, and deployment predictability without discarding the evidence-audit idea.

CC-NSIEA has important limitations. The evidence memory is internal to one benchmark, and the final study uses only the official UCI HAR training partition. The present results should therefore be interpreted as in-protocol evidence for this specific six-class smartphone-sensor setting, not as evidence of cross-dataset, device, population, or deployment generalization. The observed controller advantage is modest and requires confirmation on additional wearable activity-recognition datasets with independently defined subject-disjoint protocols. The audit rules are deliberately simple; motion-group coherence distinguishes dynamic from static activity but cannot resolve all within-group ambiguities. The τ weights are equal and the threshold is selected on finite calibration subjects. The result does not establish clinical utility, anomaly detection, streaming-event reasoning, multi-modal reasoning beyond the nine inertial channels, or generalization to a new wearable dataset. Confidence remains stronger for global AUROC and AURC, so an operational system should consider confidence and evidence audit together rather than replace one with the other. Future work should evaluate external user-disjoint datasets, investigate learned but still interpretable audit aggregation, and test how human reviewers use KPC records when a system recommends additional sensor context or manual review.

## 7. Conclusions

This paper introduced CC-NSIEA, a label-preserving neural-plus-rule-based class-contrast evidence audit for cross-subject wearable activity recognition. A Temporal Residual Perception Network supplies the fixed activity label; a training-subject evidence memory, explicit audit rules, and a finite sufficiency controller assess whether the label is supported. The method never overwrites the neural output. Under five subject-disjoint outer folds of UCI HAR, CC-NSIEA improved Error AUPRC relative to a matched dynamic deterministic controller and preserved complete KPC records for all OOF windows. The results support an evidence-centered audit complement to neural confidence, not a universal replacement for it, and not a claim of LLM-driven classification. The final design is intentionally constrained: it prioritizes traceability, matched evaluation, and an explicit boundary between perception and audit.

## Figures and Tables

**Table 1:** Shared neural perception summary.

Item	Value
OOF windows	7352
Outer-test subjects	21
Accuracy	0.901251
Macro-F1	0.905547
Accuracy 95% bootstrap CI	[0.894314, 0.908192]
Macro-F1 95% bootstrap CI	[0.899589, 0.911973]
ECE (15 bins)	0.071352
Mean confidence	0.971062
Mean entropy	0.070036
Label override permitted	False

**Table 2:** Per-class performance of the shared Temporal Residual Perception Network.

Class	Precision	Recall	F1	Support
Walking	0.971856	0.901305	0.935252	1226
Walking upstairs	0.933824	0.946878	0.940305	1073
Walking downstairs	0.979063	0.995943	0.987431	986
Sitting	0.781937	0.747278	0.764215	1286
Standing	0.784114	0.840611	0.811380	1374
Laying	0.989451	1.000000	0.994698	1407

**Table 3:** Risk-estimator comparison. CC-NSIEA is complementary to confidence rather than a universal replacement. The downward arrow indicates that lower AURC values are better.

Method	Error AUROC	Error AUPRC	AURC ↓	Top-10% Error Rate
Confidence-only	0.832074	0.390254	0.028883	0.460598
Entropy-only	0.831298	0.375512	0.028983	0.461957
Retrieval-only	0.640939	0.279624	0.069982	0.309783
Rule-only	0.553522	0.136079	0.071616	0.115489
D-NSIEA	0.786100	0.423802	0.035941	0.479620
CC-NSIEA	0.786411	0.433057	0.035913	0.479620

**Table 4:** Matched controller comparison. The LLM row is a diagnostic equivalence control, not a main method. The downward arrow indicates that lower AURC values are better.

Controller	Second-Round Policy	Error AUROC	Error AUPRC	AURC ↓
D-NSIEA	Dynamic deterministic action selection	0.786100	0.423802	0.035941
CC-NSIEA	Fixed Class-Contrast Evidence Refinement	0.786411	0.433057	0.035913
Constrained LLM diagnostic	LLM action choice; not final method	0.786411	0.433057	0.035913

**Table 5:** Subject-cluster bootstrap comparison of CC-NSIEA against D-NSIEA (10,000 resamples).

Metric	CC-D	95% Subject-Cluster CI	Fold Direction
Error AUROC	+0.000311	[0.000048, 0.000859]	4 positive; 1 equal; 0 negative
Error AUPRC	+0.009255	[0.001595, 0.019547]	4 positive; 1 equal; 0 negative
AURC (lower is better)	−0.000028	[−0.000076, −0.000004]	4 improved; 1 equal; 0 worse

**Table 6:** CC-NSIEA stopping and provenance summary.

KPC/Audit Item	Count or Value	Interpretation
sufficient_evidence	7156	97.34%
no_evidence_gain	104	1.41%
max_iter_reached	92	1.25%
Mean audit rounds	1.033324	-
KPC completeness	1.000	-
Label source	neural_temporal_resnet	All 7352 records
Label override	False	All 7352 records

## Data Availability

The UCI Human Activity Recognition Using Smartphones dataset is publicly available from the UCI Machine Learning Repository. The CC-NSIEA source code, configuration files, predeclared experimental protocol, unit tests, and execution instructions are available at: https://github.com/qzr011005-web/CC-NSIEA (accessed on 6 July 2026). The generated OOF predictions, KPC records, subject-cluster bootstrap outputs, fold-level summaries, paper tables, and execution logs are publicly available in GitHub Release v1.0.0 at: https://github.com/qzr011005-web/CC-NSIEA/releases/tag/v1.0.0 (accessed on 6 July 2026). The repository and release do not distribute the original UCI HAR data, trained checkpoints, cached outputs, or API credentials.
